# The impact of pre-existing anxiety on affective and cognitive processing of a Virtual Reality analogue trauma

**DOI:** 10.1371/journal.pone.0190360

**Published:** 2017-12-29

**Authors:** Tina Schweizer, Julian Schmitz, Laura Plempe, Dali Sun, Christian Becker-Asano, Rainer Leonhart, Brunna Tuschen-Caffier

**Affiliations:** 1 Department of Clinical Psychology and Psychotherapy, Institute of Psychology, University of Freiburg, Freiburg, Germany; 2 Department of Clinical Child and Adolescent Psychology, Institute of Psychology, University of Leipzig, Leipzig, Germany; 3 Leipzig Research Centre for Early Child Development, University of Leipzig, Leipzig, Germany; 4 Department of Artificial Intelligence, Institute of Computer Science, University of Freiburg, Freiburg, Germany; 5 Department of Social Psychology and Methodology, Institute of Psychology, University of Freiburg, Freiburg, Germany; University of Illinois at Urbana-Champaign, UNITED STATES

## Abstract

Dysfunctional processing of traumatic events may be in particular related to high trait anxiety as a pre-traumatic risk factor for the development of post-traumatic stress disorder (PTSD). However, as this has rarely been investigated in prospective, experimental studies, we aimed to analyse the association between high trait anxiety and affective as well as cognitive processing of stress using a new prospective Virtual Reality analogue trauma paradigm to overcome limitations of retrospective or current analogue designs. Individuals with high and low trait anxiety (*N* = 80) were exposed to a multi-sensory Virtual Reality emergency scenario while psychophysiological stress response, emotion regulation and intrusive memories were assessed. Our results showed that high trait anxiety individuals display increased (i) subjective stress responses, (ii) emotion dysregulation and (iii) intrusive memories upon VR analogue trauma exposure. In particular, our sample of high trait anxiety individuals displayed limited access to different emotion regulation strategies as well as increased worry and rumination regarding perceived intrusive memories. Considering the complex interplay of multiple risk factors, our findings suggests that peri-traumatic affective processing seems to mediate high trait anxiety and post-traumatic intrusive memories thereby pointing out the central role of peri-traumatic processes for intrusion development. In addition, HA as a modulating pre-traumatic risk factor might further increase the risk of later dysfunctional processing of an analogue trauma by interacting with factors of affective processing during analogue trauma exposure. Implications of these findings which may contribute to a higher risk to develop PTSD are discussed.

## Introduction

More than half of all adults are likely to be exposed to at least one traumatic event in their lifetime [1,2] defined as direct exposure to death, threatened death, actual or threatened serious injury or sexual violence, according to the Diagnostic and Statistical Manual of Mental Disorders [3]. Common initial stress responses following traumatic events are fear, helplessness, horror and subsequent involuntary intrusive memories [4,5]. While this initial stress response is only temporarily for most people [6], others fail to recover and develop stress-associated disorders such as post-traumatic stress disorder (PTSD), anxiety disorders, depression or substance abuse [7,8]. Identifying factors associated with the development of stress-associated disorders following trauma might have important prognostic and treatment implications. One key factor in this context might be individual differences in stress processing [9,10]. The aim of the current study was to explore the impact of individual differences in trait anxiety on affective and cognitive processing of an analogue trauma.

Individuals with pre-traumatic high trait anxiety (HA) may be especially vulnerable to traumatic events due to less effective stress processing. According to Spielberger’s trait-state anxiety model HA is characterised by increased neurobiological sensitivity and attention towards negative stimuli [11,12]. Thus, HA might influence affective and cognitive processing of traumatic events via more intense stress responses, emotion dysregulation and increased intrusive memories [9,13–15].

In affective processing of stress, both an intense psychophysiological stress response, and emotion dysregulation are assumed important peri-traumatic risk factors for stress-associated disorders [5,16,17]. Emotional response to analogue trauma predicts posttraumatic stress symptoms especially in individuals with high emotion dysregulation [16]. HA individuals show increased anxiety responses in general and seem to display specifically slower recovery from stress and are less able to return to initial emotional states [18,19], resulting in a specific stress response pattern including the peri-traumatic as well as recovery emotional response. Individuals with HA seem to further apply a dysfunctional pattern of emotion regulation strategies with high levels of emotional suppression and low levels of cognitive reappraisal [20,21]. The modulation of the intensity and duration of emotions is especially important in HA individuals because of their disposition to experience intense and prolonged negative emotional responses [18,22], which is associated with higher rates of psychopathology [14,23]. HA is also associated with the development of PTSD [24,25]. Thus, it is likely that individual differences in HA modulate cognitive and affective stress responses to trauma.

Impaired cognitive processing of stress is indicated by involuntary intrusive memories of the potentially traumatic event [26] as an important post-traumatic risk factor for PTSD [27]. In addition, HA seems to impair memory suppression of negative memories [28] and is associated with less fear reduction [29] and the development of intrusions [30]. In particular, persisting intrusions seem to be critical in HA individuals [9,31], contribute to the development and maintenance of PTSD [13] and constitute a core symptom of PTSD [3]. Cognitive processes modulating intrusions seems to be related to the time frame of memory consolidation, which is not exactly defined yet [32,33]. Therefore, the presumable time courses of intrusion development should be also captured in the modelling of trauma exposure and responses.

Likewise, negative appraisals of intrusive memories can elicit threat and predict PTSD [34]. According to Ehlers & Clark's cognitive model of PTSD, impaired cognitive processing due to high peri-traumatic arousal is reflected by the predominance of data-driven over conceptual processing of traumatic events, resulting in increased intrusions and disorganised trauma memories [26,35]. Particularly in HA individuals, less data-driven processing seems to result in less intrusive memories [14].

However, given that intrusive memories are also positively associated with increased emotional stress responses [30,36] as well as with emotion dysregulation [15,37], the interplay between these risk factors might be more complex. To date, only a few studies attempted to disentangle the relationship between different risk factors for maladaptive stress processing. One study revealed that the intensity of the anxious emotional response mediates the positive relationship between HA, depression, trait dissociation and subsequent intrusive memories [13]. Another study found that emotional response and pre-existing emotions predicted peri-traumatic cognitive processing, which in turn predicted emotion dysregulation. Emotion dysregulation in turn predicted intrusions [15].

The majority of studies on risk factors in coping with traumatic events have relied on retrospective assessments [5] thereby limiting their validity due to strong memory biases. Prospective analogue designs are able to overcome these limitations. One example of an established prospective analogue design in trauma research is the trauma film paradigm which comprise watching stressful films in a controlled laboratory setting [38,39]. Analogue studies have the advantage that all individuals are exposed to the same degree and type of analogue traumatic events, which allow for comparisons between individuals and to draw specific conclusions about distinct traumatic events [26]. It has further been shown that the results of clinical studies can be replicated in analogue designs with non-clinical samples [26] speaking to the external validity of analogue designs.

Prospective analogue designs for inducing and investigating stress processing in the laboratory can further be improved by advancements in Virtual Reality (VR) technology. VR extends established trauma paradigms in terms of person-environment interaction [40], and a multi-sensory simulation of an analogue trauma [41,42], both facilitating high levels of immersion and presence in VR [43]. Furthermore, in contrast to the trauma film paradigm, where perspective taking and empathising with other persons is required, the VR paradigm enables one to experience the situation and act from a first-person perspective, thereby acquiring new personal memories with high self-reference. VR provides experimental control and manipulation of distinct variables in complex situations [38,44,45]. The standardised application of VR allows for the acquisition of stress responses in real time [43] and comparing individuals while coping with stress [45,46]. An analogue trauma typically leads to qualitatively similar but attenuated psychophysiological responses as a real traumatic situation, thereby providing high ecological validity [45,46]. A previous study has been already demonstrated that the applied VR analogue trauma is as stressful as an established trauma film [47].

The aim of the current study was to test if individual differences in HA modulate affective and cognitive processing of a VR analogue trauma. We expected impaired affective processing of VR analogue trauma exposure in individuals with HA, compared to individuals with low trait anxiety (LA), indicated by increased subjective and physiological stress responses, and emotion dysregulation. Furthermore, we assumed impaired cognitive processing in individuals with HA vs. LA, indicated by increased intrusive memories after analogue trauma exposure. Considering that a more intense situational stress response as well as emotion dysregulation are also positively associated with increased intrusion development, we further expected that the peri-traumatic affective processing of stress would mediate the relationship between pre-traumatic HA and post-traumatic intrusive memories.

## Materials and methods

### Participants

In a sample of 269 healthy participants, we screened for the highest and lowest 15% extreme trait anxiety levels corresponding to the mean +/- 1 standard deviation of a normative sample of college students assessed by the State-Trait Anxiety Inventory [11,48]. A priori analysis using the g-power software revealed a sample size of 61 participants to potentially detect a between-group effect with medium effect size in a MANOVA (1-β = .80, α = .05). According to a previous study analysing a similar mediation model with adequate power [49], we aimed to collect a sample of 80 participants. Participants with sufficient language skills and without psychopharmacological medication were included in the study. Two participants were excluded from the VR analysis: one due to technical problems, the other due to nausea (cyber sickness) during data acquisition. The final sample consisted of 80 healthy participants (*N* = 80; 66 women, 14 men; age *M* = 21.86 years, *SD* = 4.14), grouped into HA and LA (*n* = 40 each). Physiological emotional arousal was investigated in a sub-sample of 40 participants in an explorative manner (*n* = 20 in each group). Participants provided written informed consent, and the study was approved by the Ethics Commission of the University of Freiburg and conducted in accordance with the Declaration of Helsinki.

### Stimuli: analogue trauma by Virtual Reality paradigm

Analogue traumatic stress was induced by a virtual and olfactory simulated emergency scenario, based on a modified video-game simulator (Valve’s Source Engine) with explicit modelling of agents' emotions. The emergency scenario was visually presented in 3-D via two colour displays of a Head Mounted Display (HMD; Type TriVisio VR Vision), auditorily via headphones and olfactorily by exuding the smell of smoke via a ventilator to ensure multi-sensory simulation [41,42]. A Calibri tracker transmitted participants’ head movements in the HMD to register visual field changes within the VR. Movements within the VR were performed by using a joystick. We assessed and controlled for previous experience with computer games. To adapt to the VR environment and to learn how to navigate and act using the HMD and joystick, a neutral training scenario in the same environment as in the experimental condition but without any negative events was simulated before the analogue trauma scenario. The analogue trauma scenario consisted of an unexpected emergency situation simulated in an underground parking lot. Participants were instructed to go to their car in the virtual parking garage. Shortly before reaching the car, a loud detonation followed by fading lights was presented and a smell of smoke was atomised. Psychological and physical threat was induced by the presentation of an increasing amount of smoke and coughing sounds. An adjacent burning car as well as an injured man trapped under some objects and crying for help was displayed. Within the scenarios, participants were able to interact with the environment from a first-person perspective. The game engine automatically registered the time within the VR scenarios and finished them as soon as the participants left the virtual parking garage.

### Demographic and psychometric measures

Demographic characteristics, experience with computer games and previous potentially traumatic events (referring to a study from Foa et al. [50]) were assessed prior to the experiment. The sense of presence as well as the interaction within VR were examined after the experiment on an 11-point Likert scale (ranging from 0 = *not at all* to 10 = *very intensely*) [51].

Trait anxiety was measured using the State-Trait Anxiety Inventory (STAI-T) which shows good internal consistency (*α* = .90) and retest-reliability (*r* = .77 to .90) [11,48].

Depressive symptoms occurring in the two weeks before the experiment were assessed using the revised Beck Depression Inventory (BDI II) demonstrating good internal consistency (*α* = .90) and retest-reliability (*r* = .78) [52,53].

Subjective anxiety was rated on an 11-point Likert intensity scale (ranging from 0 = *very low* to 10 = *very strong*) [51].

Emotion dysregulation was measured with the 36-item Difficulties in Emotion Regulation Scale (DERS), containing six subscales: non-acceptance, goals, impulse, awareness, strategies, and clarity. The DERS has good internal consistency (*α* = .81 - .95) and satisfactory retest-reliability (r = .77 - .87) [54].

Spontaneous involuntary intrusive memories of pictures, sounds and thoughts were assessed by the modified 10-item Intrusive Memory Questionnaire [55] concerning the frequency (referring to the absolute number of occurrences via a numeric scale indicating the absolute numbers including “0”) and temporal occurrence (referring to the relative amount of time in which specific intrusive memories occurred via a percentage scale ranging from 0–100 (%) indicating the relative amount of time of the total time) of intrusive memories, perceived worry about intrusive memories (via a decimal step scale ranging from “0” = *not present* to “100” = *most extreme*) and general mental occupation with the experienced analogue trauma (via a percentage scale ranging from 0–100 (%) as the relative amount of time of the total time). We directly combined items to group sensory modalities such as (i) frequency of pictures and sounds and (ii) temporal occurrence of pictures and sounds. The frequency and temporal occurrence of thoughts and the general mental occupation with the event as cognitive entities were initially separately depicted. In addition, we directly combined all items related to worry (regarding intrusive pictures, thoughts and sound) as a temporally subsequent appraisal. In the current study internal consistency was *α* = .81 - .87.

### Acquisition and analysis of the physiological data

We recorded SCL as an objective biological marker and sensitive indicator of emotional arousal [56]. Since SCL is regulated by the sympathetic nervous system, it is strongly associated with emotional processes [57]. As SCL is also sensitive to changes in temperature and noise, the experiment was performed in a quiet, temperature-controlled room (22°C). Physiological data were recorded at 400 Hz by Varioport II Systems (Becker Meditec, Karlsruhe, Germany). SCL was conducted at a sampling rate of 125 Hz over 11 mmAg/AgCl electrodes, filled with isotonic paste, over the middle phalanx of the index and middle fingers of the immobilised non-dominant hand. A constant current flow voltage of 0.5 V was applied by two electrodes. SCL raw signals were smoothened by a 1 Hz low-pass filter and controlled during conduction. Participants were instructed to avoid movements and were monitored during the experiment for movement artefacts. The SCL raw data were natural log transformed (Ln(SCL)) to normalise the data [56].

### Procedure

We used a mixed design with trait anxiety (low vs. high) as between subject factor and condition (VR analogue trauma and recovery phase 5 minutes after the stress induction) as within subject factor. Additionally, a one-day follow-up assessment of intrusive memories was performed. Before the experiment, baseline levels of the emotional states were obtained and all participants completed a VR training. At the beginning of the experiment, participants received general information about the study and completed questionnaires regarding socio-demographic data, computer game experience and previous potentially traumatic events on a desktop PC. In our study, all instructions were standardised. During the baseline, participants watched a non-arousing five-minute film clip of landscapes. Afterwards, participants adapted and practiced VR in the training scenario. Participants were then exposed to the analogue trauma. In the subsequent recovery phase, participants were instructed to sit calmly and relax for 5 min in order to assess recovery from stress. After each phase, subjective intensity of anxiety was assessed. Physiological emotional arousal was recorded continuously throughout the experiment via electrodermal activity by SCL amplitudes. Emotion dysregulation was measured by DERS in respect to the VR analogue trauma. Furthermore, participants evaluated the sense of presence as well as the interaction within the VR simulation. Finally, participants completed the IMQ. Participants completed the IMQ again at the following day, and then received a debriefing. The IMQ was applied at two time points to capture also the time course of intrusive memories. The time period between experiment and the follow-up investigation of the IMQ were identical in both groups.

### Data processing and statistical analyses

Peripheral physiological data parameters (SCL) were analysed using ANSLAB at one minute intervals that were further averaged over 3 min for each phase [58].

An index sum score of all 6 partly combined intrusive memory items from the modified IMQ (therefore indirectly including all 10 items from the original IMQ) was calculated by using z-standardisation in order to make different answer rating scales comparable and facilitate their interpretation on a standard normal distribution. To analyse the single IMQ parameters in more detail, we used the 4 main dimensions of the IMQ as depended variables including all respective items (frequency, temporal occurrence, worry, general mental occupation).

Group differences on subjective and physiological stress response between HA and LA upon VR analogue trauma exposure were tested by one-way repeated measures analyses of covariance (ANCOVAs) with group as the between factor (HA, LA) and condition (analogue trauma, recovery) as the within factor. Baseline levels of the dependent variables were included as covariates. Group differences regarding emotion dysregulation of HA and LA were analysed by a one-way multivariate analysis of variance (MANOVA) for DERS subscales. To test for group differences between HA and LA regarding intrusive memories a one-way repeated measure ANOVA for the IMQ index score as well as a MANOVA for the IMQ single parameters were conducted with group as the between factor (HA, LA) and the two time points when intrusive memories were measured (5 min after the VR analogue trauma and at 9 pm the next day) as the within factor. After a significant multivariate overall test, the univariate main effects were described. No post hoc analyses were performed since all independent variables included in the MANOVA only had two levels [59].Data analyses were performed using SPSS version 21.0. In order to investigate the relationship between trait anxiety and intrusive memories, mediation analysis based on structural equation modelling (SEM) was conducted using Mplus Version 7.3 [60] which allows for the testing of models with categorical predictors. Effect sizes η^2^ (small ≥ 0.01; moderate ≥ 0.06; large ≥ 0.14), *d* (small ≥ 0.2; moderate ≥ 0.5; large ≥0.8) and *r* (small ≥ 0.1; moderate ≥ 0.3; large ≥ 0.5) were calculated and classified according to Cohen [61]. A significance level of α = .05 was chosen for two-tailed conservative hypothesis testing.

## Results

### Participants

The HA group had more depressive symptoms and a higher proportion of females than the LA group. The groups did not differ on age, previous traumatic events, computer game experiences or exposure times within the virtual emergency scenario. (**Table 1**)

**Table 1:** Participant characteristics.

	Trait anxiety		Test statistic	*p*
	low (*n* = 40)	high (*n* = 40)		
Sex: *N* (%)			χ^2^(1, *N* = 80) = 5.54	.019*
female	29 (72.5)	37 (92.5)		
male	11 (27.5)	3 (7.5)		
Age (years): *M (SD)*	21.75 (4.81)	21.98 (3.39)	*t*(78) = 0.24	.810
STAI-T: *M (SD)*	28.64 (2.03)	53.20 (6.13)	*t(78) =* 24.03	< .001***
BDI-II: *M (SD)*	2.46 (2.86)	7.87 (3.15)	*t*(78) = 7.95	< .001***
Previous traumatic events: *M (SD)*	1.10 (1.22)	1.03 (1.37)	*t*(78) = 0.26	.796
Experience playing computer games: %	32.5	32.5	χ^2^(1, *N* = 80) = .000	.100
Exposure time (Min) analogue trauma: *M (SD)*	4.86 (2.90)	4.42 (1.60)	*t*(74) = 0.81	.418

*Note*. STAI-T = State-Trait Anxiety Inventory; BDI-II = Beck Depression Inventory-II; Previous traumatic events were assessed referring to a study from Foa et al [50].

*p < .05. **p< .01. ***p < .001.

### Manipulation check

Paired sample t-tests between baseline and the analogue trauma phase were conducted to validate the stress induction. There was a significant increase with large effect sizes from baseline to VR analogue trauma for subjective anxiety (*t*(78) = 15.69, *p* < .001, *d* = 1.77) and physiological emotional arousal (*t*(37) = 11.69, *p* < .001, *d* = 1.90), indicating that the experimental manipulation was successful. The two groups did not differ in the level of presence (t(78) = 1.29, p = .206; *M*_HA_ = 7.28, *SD*_HA_ = 1.48; *M*_LA_ = 6.66, *SD*_LA_ = 1.51) or interactivity within the VR scenario (t(78) = 1.95, p = .059; *M*_HA_ = 3.58, *SD*_HA_ = 2.82; *M*_LA_ = 5.34, *SD*_LA_ = 2.85).

### Trait anxiety and psychophysiological stress response

Baseline levels of the dependent variables were included as covariates, with no between-group differences in baseline levels of subjective anxiety (t(76) = 0.40, p = .689) nor physiological emotional arousal (t(36) = 0.77, p = .447).

The 2 (group: HA, LA) x 2 (time: analogue trauma, recovery) ANCOVA for subjective anxiety revealed a significant main effect of group (*F*(1, 75) = 9.00; *p* = .004, η_p_^2^ = 0.107) and time (*F*(1, 75) = 174.03; *p* < .001, η_p_^2^ = 0.699), but no interaction between group x time (*F*(1, 75) = 0.14; *p* = 0.905). Participants with HA scored higher on subjective anxiety than participants with LA in both the analogue trauma and recovery phase. Both groups scored higher on subjective anxiety in the analogue trauma compared to the recovery phase. The baseline level of subjective anxiety was significantly related to subjective anxiety under stress and recovery (*F*(1, 75) = 7.75; *p* = .007, η_p_^2^ = 0.094).

Furthermore, the 2 (group: HA, LA) x 2 (time: analogue trauma, recovery) ANCOVA for physiological emotional arousal assessed by SCL in a sub-sample of *n* = 20 per group revealed no significant main effect of group (*F*(1, 35) = 0.05; *p* = .830), however, a significant main effect of time (*F*(1, 35) = 8.75; *p* = .006, η_p_^2^ = .200). The interaction for time x group was not significant (*F*(1, 35) = 2.02; *p* = .164). Participants in both groups demonstrated a higher SCL during the analogue trauma compared to the recovery phase. The baseline level of SCL was significantly related to physiological emotional arousal under stress and recovery (*F*(1, 35) = 51.83; *p*< .001, η_p_^2^ = 0.597).

### Trait anxiety and peri-traumatic emotion dysregulation

**Fig 1:**
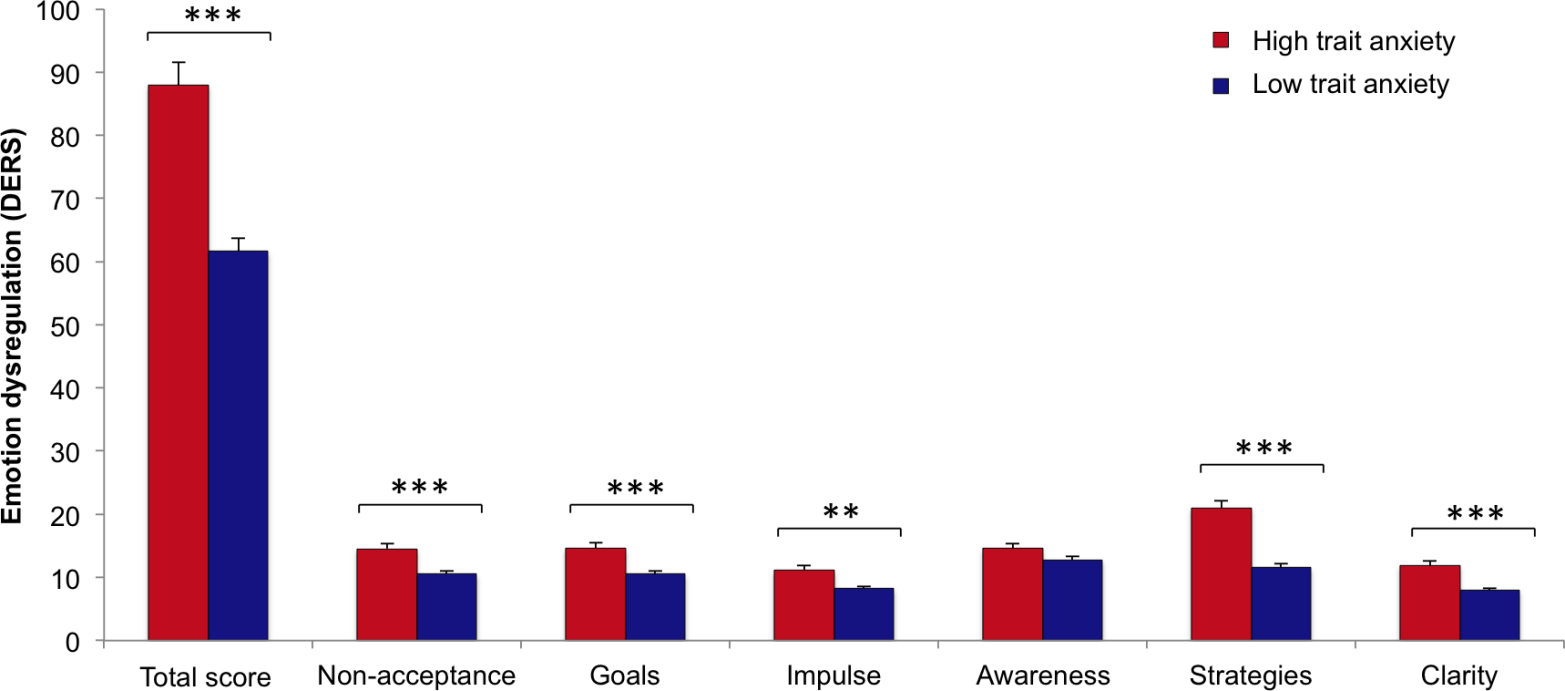
Emotion dysregulation of a Virtual Reality analogue trauma as a function of trait anxiety. Group differences between high and low trait anxiety regarding emotion dysregulation are shown, as measured by the total score as well as the subscales of the difficulties in emotion regulation scale (DERS). Between group differences of high and low trait anxious individuals are indicated (**p< .01; ***p< .001). Error bars represent standard errors of the mean.

The one-way MANOVA for emotion dysregulation revealed a significant main effect of group (*F*(1, 78) = 10.39, *p*< .001, η_p_^2^ = .461), indicating higher levels of emotion dysregulation in the HA compared to the LA group. Increased levels of emotion dysregulation were found in the HA group for the subscales *non acceptance of emotional responses* (*F*(1,78) = 18.81, *p* < .001, η_p_^2^ = .194), *difficulties engaging in goal directed behaviour* (*F*(1,78) = 19.62, *p* < .001, η_p_^2^ = .201), *impulse control difficulties* (*F*(1,78) = 11.13, *p* = .001, η_p_^2^ = .125), *limited access to emotion regulation strategies* (*F*(1,78) = 54,32, *p* < .001, η_p_^2^ = .411) and *lack of emotional clarity* (*F*(1,78) = 24.88, *p* < .001, η_p_^2^ = .242). In contrast, no significant difference was found for the *lack of emotional awareness* subscale *(F*(1, 78) = 3.76, *p* = .056). **(Fig 1)**

### Trait anxiety and post-traumatic intrusive memories

**Fig 2:**
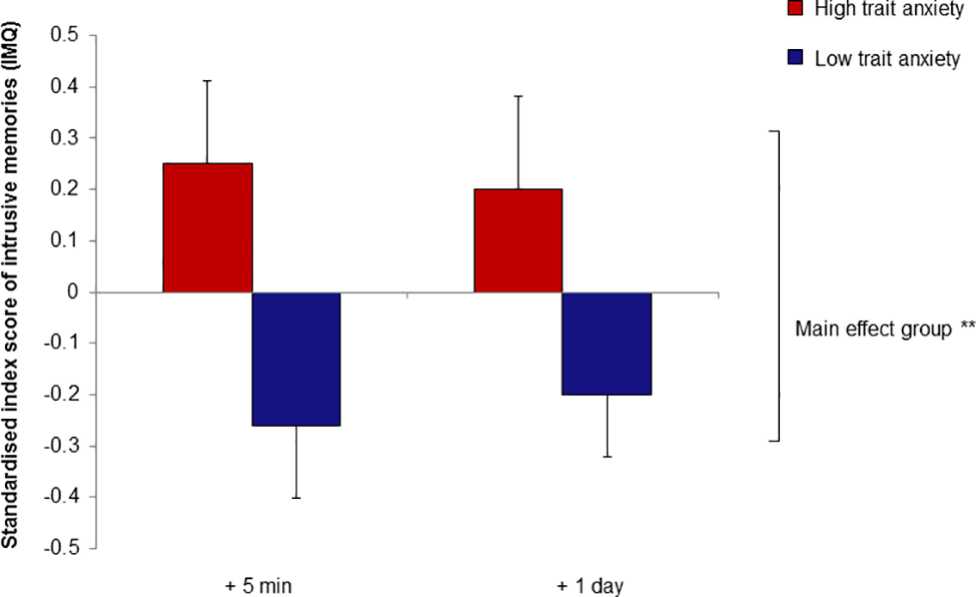
Intrusive memories after a Virtual Reality analogue trauma as a function of trait anxiety. The standardised index score of subsequent intrusive memories is displayed for five minutes afterwards and the following evening at 9 pm, as measured by the Intrusive Memory Questionnaire (IMQ). Between group differences of high and low trait anxious individuals are indicated (**p< .01;). Error bars represent standard errors of the mean.

A one-way repeated measure ANOVA regarding the IMQ index score revealed a statistically significant main effect of group (*F*(1, 76) = 9.79, *p* = .002, η_p_^*2*^ = .114), but not for time (*F*(1, 76) = .005, *p* = .947) nor for the interaction between time x group (F(1, 76) = .447, *p* = .506). Participants in the HA group displayed increased intrusive memories at both time points (5 min after the VR analogue trauma and at 9 pm the next day) in comparison to the LA group. (**Fig 2**).

To analyse the single IMQ parameters in more detail we additionally conducted a one-way repeated measure MANOVA. Beside the significant multivariate effect of group on the dependent variables related to intrusive memories (*F*(4,73) = 3.23, *p* = .017, η_p_^2^ = .150), a significant main effect of group was found concerning the frequency (*F*(1,76) = 5.01, *p* = .028, η_p_^2^ = .062) and temporal occurrence of intrusive memories (*F*(1,76) = 7.71, *p* = .007, η_p_^2^ = .092) as well as perceived worry about intrusive memories (*F*(1,76) = 11.41, *p* = .001, η_p_^2^ = .131) and general mental occupation with the experienced analogue trauma (*F*(1,76) = 4.89, *p* = .030, η_p_^2^ = .060). Furthermore, we found a significant multivariate effect of time concerning the intrusive memories variables (*F*(4, 73) = 58.62, *p* < .001, η_p_^2^ = .763). No significant interaction effects between time x group were observed (*F*(4, 73) = 1.33 *p* = .266). At both time points (t1 = 5 min after the VR analogue trauma; t2 = 9 pm the next day), the HA group displayed higher values on the IMQ single parameters than the LA group. At t1 both groups displayed higher values than at t2 for the frequency (*F*(1, 76) = 60.67, *p* = .001, η_p_^2^ = .444) and temporal occurrence of intrusive memories (*F*(1, 76) = 123.89, *p* < .001, η_p_^2^ = .620), worry about intrusive memories (*F*(1, 76) = 117.55, *p* < .001, η_p_^2^ = .607) and general mental occupation with the analogue trauma (*F*(1, 76) = 198.28, *p* < .001, η_p_^2^ = .723).

### Relationship between trait anxiety and intrusive memories

**Fig 3:**
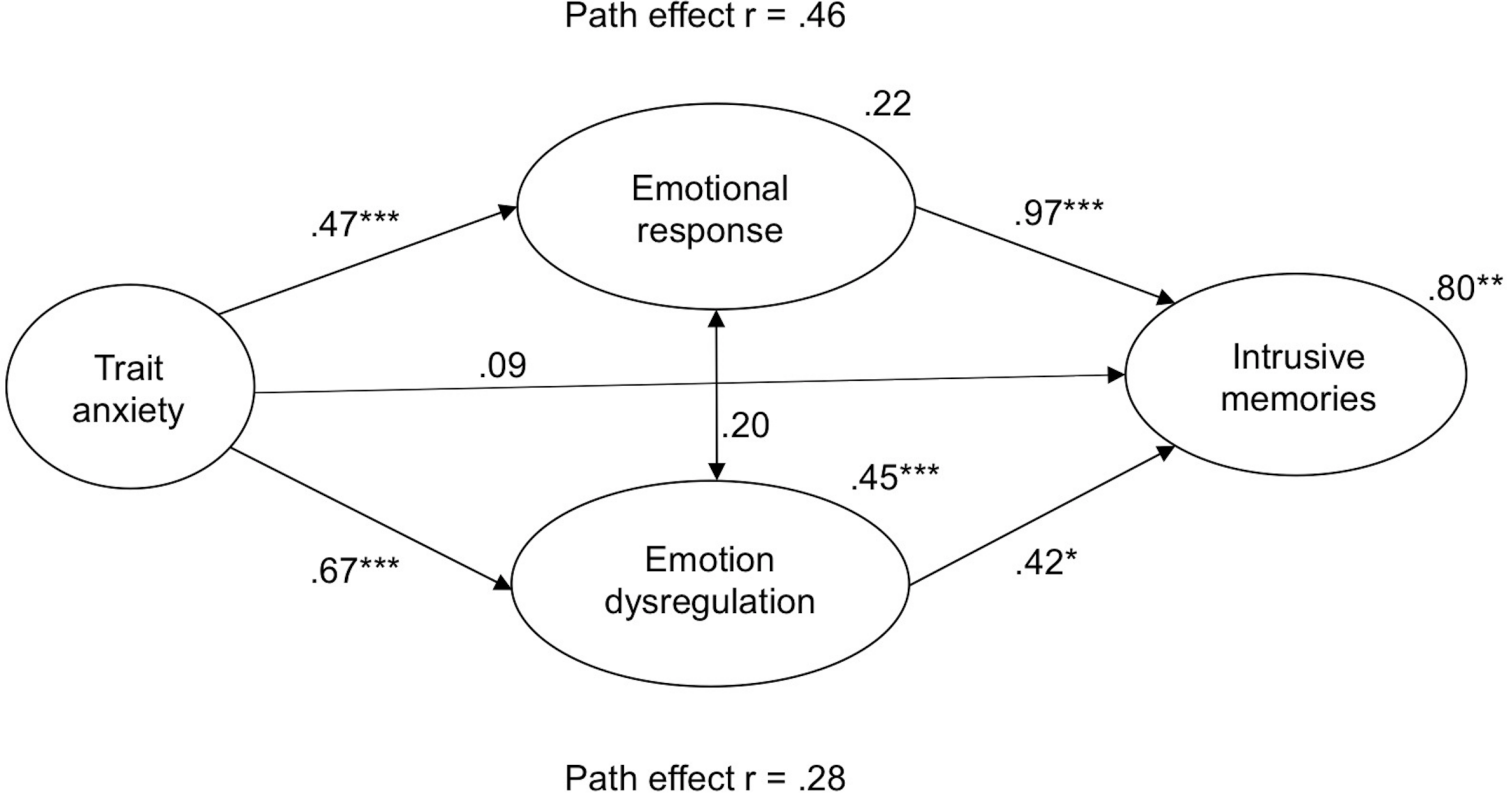
Mediation analysis of the relationships between trait anxiety, emotional response, emotion dysregulation and intrusive memories after a Virtual Reality analogue trauma. (Chi^2^/df = 106.214/68; RMSEA = .085; CFI = .934; TLI = .911). Note: Standardized path coefficients are depicted for the direct effects between pre-traumatic trait anxiety and peri-traumatic emotional response of anxiety and emotion dysregulation as well as post-traumatic intrusive memories. The explained variances are shown for emotional response, emotion dysregulation and intrusive memories. Path effects indicate the mediated relationship between trait anxiety and intrusive memories by emotional response (see at the top) as well as emotional dysregulation (see below).

A mediation analysis based on SEM was conducted to investigate the relationship between trait anxiety and intrusive memories. The evaluation of the model fit was performed with the root mean square error of approximation (RMSEA), the comparative fit index (CFI) and the Tucker–Lewis index (TLI), as recommended fit indices [62]. Results indicated that the direct paths from trait anxiety to peri-traumatic emotion dysregulation (β = .67, *p* < .001) and emotional response of anxiety were significant (β = .47; *p* < .001) with an accounted variance of 45% for emotion regulation (*p* < .001) and of 22% for emotional response (*p* = .08). Additionally, the direct paths between emotion dysregulation and intrusive memories (β = .42; *p* = .02) as well as between emotional response of anxiety and intrusive memories (β = .97; *p* < .001) were significant. In the more complex model in which two potential mediators were added, the direct path between trait anxiety and intrusive memories was no longer significant (β = .09, *p* = .62) in predicting intrusive memories following analogue trauma. Moreover, there were significant indirect path effects between trait anxiety and intrusive memories mediated by emotional response of anxiety (*r* = .46) and dysregulation (*r* = .28). The hypothesised mediation model (Chi^2^/df = 106.214/68, RMSEA = .085, CFI = .934, TLI = .911) fulfilled all recommended fit indices [62] and accounted for 80% (*p* < .01) of the variance in intrusive memories. **(Fig 3)**

## Discussion

This study tested if individual differences in HA modulate affective and cognitive processing of a VR analogue trauma. We expected that in an analogue trauma, individuals with HA would show impaired affective and cognitive processing of stress, compared to individuals with LA. Our main finding in a novel VR analogue trauma paradigm was that HA negatively modulates affective and cognitive processing of stress indicated by increased subjective stress responses, emotion dysregulation and intrusive memories. In particular, individuals with HA displayed limited access to different emotion regulation strategies as well as increased worry and rumination regarding perceived intrusive memories. Furthermore, affective processing seems to mediate HA and intrusive memories.

A major strength of our study was that the applied prospective analogue design prevents memory biases and allows for an ethically appropriate, controlled experimental setting [38] including a moderate VR stress induction that has been proven as stressful as an established trauma film [47]. In our study, participants generally rated the presence and interactivity within the VR scenario as medium to high. Likewise, cyber sickness was not a problem: Only one participant felt nauseous from undertaking the VR (and were then excluded from the analysis).

Our findings revealed that VR analogue trauma exposure leads to higher subjective anxiety in HA individuals than LA individuals in line with Spielberger’s trait-state anxiety model [11] and with previous studies [e.g.13]. Previous studies have found that individuals with HTA showed a higher risk to develop PTSD, which was classified as anxiety disorder until the change to DSM-5 into a distinct diagnostic group of trauma and stressor-related disorders [2,3,24]. Although intense peri-traumatic negative emotions which we have found in our study are considered a natural first stress response upon confrontation with a potentially traumatic event [4,17], increased peri-traumatic anxiety is associated with a higher risk of developing stress-associated symptoms [14] as well as higher peri-traumatic stress is associated with an increased risk to develop PTSD [63]. The persisting stronger subjective emotional response in individuals with HA during and after the VR analogue trauma suggests that individuals with HA have problems downregulating the experienced anxiety. This is in line with previous studies showing increased difficulties returning to initial emotional states after negative emotions in participants with anxiety symptoms [18] and the result of higher anxiety symptoms in patients with PTSD [64,65]. Physiological stress responses did not differ between the groups, which is in contrast to our expectation and a previous fMRI study showing increased skin conductance responses to unpredictable threat in individuals who were exposed to stressful events and in patients with PTSD [66]. Our finding that there is no difference in physiological stress responses between HA and LA may be related to (i) a blunted hormonal and skin conductance response to acute stress found to be specific for HA individuals [67,68], (ii) efficient coping behaviour [69] or (iii) the fact that analogue traumatic situations are less intense than real traumatic situations [45]. Another explanation could be a lack of statistical power in this analysis of a smaller sub-sample.

An impaired capacity to regulate and cope with highly intense emotions leads to the maintenance of strong emotions and may function as an important peri-traumatic risk factor for stress-associated disorders [16,17]. In our study, HA individuals generally demonstrated increased emotion dysregulation, which is in line with previous non-clinical [18] and clinical [70,71] studies. In particular, our sample with HA showed more difficulties in understanding, accepting and regulating their negative emotions as well as controlling impulses and focusing on goal-directed behaviour. Among those, the most severe problem in emotion dysregulation, both within the HA group as well as compared to the LA group, was limited access to emotion regulation strategies, which is considered to be particularly maladaptive [6,72]. Since our VR analogue trauma provided potential situation controllability, goal-directed behaviour was considered the most adaptive emotion regulation strategy referring to the concept of situation modification according to the process model of emotion regulation [73] as well as problem-focused coping within the transactional model of stress and coping [74]. In other healthy samples, HA individuals also applied fewer adaptive emotion regulation strategies [72] and reported more difficulties in understanding, accepting and regulating negative emotions [18]. In another non-clinical sample, emotion dysregulation before a traumatic event predicted the severity of later intrusive memories, more intense emotional responses and perceived threat to a traumatic stressor [17]. In clinical samples, emotion dysregulation has also been associated with PTSD, with a lack of emotional acceptance and emotional clarity, difficulties in impulse control and engaging in goal-directed behaviour [75].

In the overall sample intrusive memories were increased directly after the analogue trauma compared to one day later in line with the finding that intrusive memories after trauma decline in intensity and frequency over time [44,76]. HA individuals displayed increased and remaining intrusive memories also one day after analogue trauma. This is in accordance with previous findings [9,13,14,31], showing that HA is related to increased intrusive memories as a consequence of impaired cognitive processing of stress [26] and to persisting intrusive memories, which have been found to be particularly dysfunctional and associated with PTSD [13,77,78]. This might be due to impaired cognitive control found in HA individuals via a decreased suppression of negative memories [28] and reduced prefrontal attentional top-down regulation in the inhibition of distraction processes [79]. Patients with PTSD displayed also an impaired working memory performance, but this seems additionally influenced by anxiety, depression and experienced stress exposure [65]. However, we do not investigate this explicitly.

Likewise, worry and mental occupation about intrusive memories were higher directly after the VR analogue trauma compared to one day later in the overall sample. HA individuals showed increased worry and mental occupation, which remained at a high level one day after analogue trauma exposure. This is in line with findings showing that HA is associated with worry [80] and maladaptive mental occupation [81]. Particularly, previous studies have shown that individuals with HA and a high level of worry tend to ruminate [15,35,77]. The persistence of worry and mental occupation in HA individuals might be particularly critical for maladaptive stress processing following analogue trauma [34,35,82]. Both maintain PTSD symptoms and can function as cognitive avoidance of emotions thereby increasing the risk of PTSD development [27,31,36,77].

Considering the complex interplay of multiple risk factors in coping with high-stress, our findings are in line with previous studies [13,15] and extend these by using a prospective VR analogue trauma paradigm. Our results suggest that peri-traumatic affective stress processing mediates the pre-traumatic risk factor HA and subsequent intrusive memories via an increased subjective emotional response and emotion dysregulation. Furthermore, we found direct effects of peri-traumatic emotional response and emotion dysregulation on intrusion development. Hence, our findings highlight the central role of peri-traumatic processing in intrusion development. This is in line with studies showing that both, emotional response and regulation, predict post-traumatic stress symptoms such as intrusive memories [15,16,69]. Emotion dysregulation seems to block the effective regulation of anxiety [83]. Moreover, the association of increased emotional stress responses and post-traumatic stress symptom severity was found only in the context of higher levels of emotion dysregulation [16]. Furthermore, also other studies highlighted the central role and importance of emotion dysregulation for developing post-traumatic stress symptoms by the finding that emotion dysregulation mediates between the experience of childhood abuse and later post-traumatic stress symptoms [84,85]. The relation between emotion dysregulation in response to traumatic events and the development of PTSD symptoms might be further related to the applied emotion regulation strategies, e.g. PTSD symptoms were associated with more suppression and less reappraisal [69]. At the neurobiological level, emotional dysregulation with less application of adaptive strategies such as reappraisal may result from a reduced connectivity between the amygdala and the prefrontal cortex (PFC) [86] especially in HA individuals [87]. Reappraisal as a cognitive emotion regulation strategy results in decreased amygdala and increased prefrontal activity [88]. Hence, emotion regulation as potentially modifiable risk factor seems to be most important as target for interventions. Reappraisal training, for example, can lead to less frequent intrusions and decreased stress responses [89]. In addition, our results suggest the importance of pre-traumatic anxiety in increasing later dysfunctional peri-traumatic affective processing, which in turn may contribute to an impaired cognitive processing of stress. Thus, our findings suggest that pre-traumatic high trait anxiety may additionally interact with affective processing during analogue trauma exposure to confer risk for the development of stress-associated disorders. This is consistent with the diathesis stress model of PTSD development [90,91].

### Limitations

Individuals with HA in this study reported higher depressive symptoms compared to those with LA, which is in line with previous studies describing high comorbidity due to symptoms’ overlap in both non-clinical [92] as well as clinical samples [93]. However, on average the degree of depressive symptoms in our sample was rather low in both groups. Nevertheless, we would recommend that future studies also focus on depressive symptoms due to their potential relation to intrusive memories [94].

Although only valid self-report questionnaires were used, subjective evaluation of emotion regulation capacities might be confounded by reflection capability [95]. We further examined emotion dysregulation using a modified established trait questionnaire since no state instrument exists. However, it has been shown that emotion regulation is required particularly in stressful situations [6]. In the current study we did not focus on how peri-traumatic responses separately affects the occurrence and related subsequent appraisals of intrusive memories, which could be of interest for future studies. No measures of cognitive or attentional control were applied. Additionally, we did not ask for previous life events, although we did assess previous potentially traumatic events.

Since we used one specific stress situation of an emergency in the VR environment, the results might therefore differ for other stress situations.

### Implications

Our findings have potential clinical implications and highlight the importance of identifying vulnerable individuals at risk of maladaptive but modifiable stress processing [96]. In the sense of primary prevention [96], they should be protected from high-stress situations (e.g. by personnel selection) or prepared to high-stress situations prior to exposure thereby preventing mental health problems or mental disorders [44]. As prophylactic measures to mitigate the development of PTSD in individuals with high trait anxiety we propose to particularly assess problems in emotion regulation (e.g. via DERS) as important peri-traumatic modulator as well as coping flexibility (e.g. via the Perceived Ability to Cope with Trauma (PACT) Scale; [97]) as probably the most adaptive regulation style in coping with stress [98,99]. Individuals that were occupationally high-stress exposed (e.g. emergency forces, police services, soldiers) or scoring high on trait anxiety might benefit from emotion regulation training, particularly regarding flexible access and application of different context-appropriate emotion regulation strategies [6,72]. Acceptance-based approaches [100] could improve coping with persistent worrying and rumination by changing negative appraisals about intrusive memories and reducing avoidance.

Future studies should further analyse the complex relations of multiple risk factors for a better understanding of the development of stress-associated disorders. In addition, other VR analogue stress situations should be developed to compare different types of stressful situations and behavioural measures should be integrated.

The VR analogue trauma paradigm may contribute to prevent stress-associated disorders in the future by interventions targeting peri- and post-traumatic risk factors in real-time.

## Conclusions

In conclusion, this is the first study to investigate the complex interplay of multiple risk factors in stress processing in a prospective VR analogue trauma paradigm. Our findings suggest that HA contributes to dysfunctional affective and cognitive processing of a VR analogue trauma. In particular, our HA sample displayed limited access to different emotion regulation strategies as well as increased worry and rumination regarding perceived intrusive memories. Peri-traumatic affective stress processing seems to mediate the pre-traumatic risk factor HA and subsequent intrusive memories via an increased subjective emotional response and emotion dysregulation. Hence, our findings point out the central role of peri-traumatic processing in intrusion development. Furthermore, our findings suggest the importance of pre-traumatic anxiety in increasing later dysfunctional peri-traumatic affective processing, which in turn may contribute to an impaired cognitive processing of an analogue trauma. Thus, based on our results, pre-traumatic risk factors such as high trait anxiety may additionally interact with factors during analogue trauma exposure to confer risk for the development of stress-associated disorders. Our results thereby contribute to a better understanding of individual modulating mechanisms in coping with high stress situations and provide important information for the theoretical modelling of PTSD development. The innovative prospective VR analogue trauma paradigm is a substantial methodological advancement in analogue designs, offering an effective and ethically acceptable method to induce stress experimentally in a standardised laboratory setting. As a novel experimental psychopathology model for studying trauma exposure and responses, it can contribute to future prevention and treatment development.

## Supporting information

S1 Fig Screenshots from the Virtual Reality analogue trauma. (TIF)

S1 Table Variables of affective and cognitive processing of an analogue trauma. *Note*. t1 = 5 min after the VR analogue trauma; t2 = 9 pm the next day. Combined items of the modified version of the intrusive memory questionnaire (IMQ): (i) frequency of pictures and sounds, (ii) temporal occurrence of pictures and sounds to group sensory vs. cognitive modalities; (iii) worry about intrusive pictures, thoughts and sounds as subsequent appraisal.
(PDF)

## References

[1] Kessler RC, Sonnega A, Bronet E, Hughes M, Nelson CB. Posttraumatic stress disorder in the national comorbidity survey. Arch Gen Psychiatry. 1995;52(10):1048–60. 7492257 10.1001/archpsyc.1995.03950240066012

[2] Kilpatrick DG, Resnick HS, Milanak ME, Miller MW, Keyes KM, Friedman MJ. National estimates of exposure to traumatic events and PTSD prevalence using DSM-IV and DSM-5 criteria. J Trauma Stress [Internet]. 2013 10 [cited 2016 Feb 26];26(5):537–47. Available from: http://www.pubmedcentral.nih.gov/articlerender.fcgi?artid=4096796&tool=pmcentrez&rendertype=abstract 10.1002/jts.21848 24151000 PMC4096796

[3] American Psychiatric Association. Diagnostic and statistical manual of mental disorders (5th ed). Arlington, VA: American Psychiatric Publishing; 2013 991 p.

[4] Bernat J, Ronfeldt H, Calhoun K, Arias I. Prevalence of traumatic events and peritraumatic predictors of posttraumatic stress symptoms in a nonclinical sample of college students. J Trauma Stress. 1998;11(4):645–64. 10.1023/A:1024485130934 9870219

[5] Ozer EJ, Best SR, Lipsey TL, Weiss DS. Predictors of posttraumatic stress disorder and symptoms in adults: A meta-analysis. Psychol Bull. 2003;129(1):52–73. 12555794 10.1037/0033-2909.129.1.52

[6] Bonanno GA, Westphal M, Mancini AD. Resilience to loss and potential trauma. Annu Rev Clin Psychol. 2011;7:511–35. 10.1146/annurev-clinpsy-032210-104526 21091190

[7] Bryant RA, Friedman MJ, Spiegel D, Ursano R, Strain J. A review of acute stress disorder in DSM-5. Depress Anxiety. 2011;28(9):802–17. 10.1002/da.20737 21910186

[8] Shalev A, Sahar T, Freedman S, Peri T, Glick N, Brandes D, et al A prospective study of heart rate response following trauma and the subsequent development of posttraumatic stress disorder. Arch Gen Psychiatry. 1998;55(6):553–9. 9633675 10.1001/archpsyc.55.6.553

[9] Tampke AK, Irwin HJ. Dissociative processes and symptoms of posttraumatic stress in vietnam veterans. J Trauma Stress [Internet]. 1999;12(4):725–38. Available from: http://www.ncbi.nlm.nih.gov/pubmed/10646190 10.1023/A:1024733621595 10646190

[10] Weems CF, Pina AA, Costa NM, Watts SE, Taylor LK, Cannon MF. Predisaster trait anxiety and negative affect predict posttraumatic stress in youths after hurricane Katrina. J Consult Clin Psychol [Internet]. 2007;75(1):154–9. Available from: http://www.ncbi.nlm.nih.gov/pubmed/17295574 10.1037/0022-006X.75.1.154 17295574

[11] Laux L, Glanzmann P, Schaffner P, Spielberger C. Das State-Trait-Angstinventar (STAI) [State-Trait-Anxiety Inventory (STAI)] Weinheim, Germany: Beltz; 1981.

[12] O’Donovan A, Slavich GM, Epel ES, Neylan TC. Exaggerated neurobiological sensitivity to threat as a mechanism linking anxiety with increased risk for diseases of aging. Neurosci Biobehav Rev [Internet]. 2013;37(1):96–108. Available from: 10.1016/j.neubiorev.2012.10.013 23127296 PMC4361087

[13] Laposa JM, Alden LE. The effect of pre-existing vulnerability factors on a laboratory analogue trauma experience. J Behav Ther Exp Psychiatry [Internet]. 2008;39(4):424–35. Available from: 10.1016/j.jbtep.2007.11.002 18294615

[14] Logan S, O’Kearney R. Individual differences in emotionality and peri-traumatic processing. J Behav Ther Exp Psychiatry [Internet]. 2012;43(2):815–22. Available from: http://linkinghub.elsevier.com/retrieve/pii/S0005791611001285 10.1016/j.jbtep.2011.12.003 22197753

[15] Regambal MJ, Alden LE. Pathways to intrusive memories in a trauma analogue paradigm: A structural equation model. Depress Anxiety. 2009;26(2):155–66. 10.1002/da.20483 19217072

[16] Badour CL, Feldner MT. Trauma-related reactivity and regulation of emotion: Associations with posttraumatic stress symptoms. J Behav Ther Exp Psychiatry [Internet]. 2013;44(1):69–76. Available from: 10.1016/j.jbtep.2012.07.007 22922079 PMC3508380

[17] Bardeen JR, Kumpula MJ, Orcutt HK. Emotion regulation difficulties as a prospective predictor of posttraumatic stress symptoms following a mass shooting. J Anxiety Disord. 2013;27(2):188–96. 10.1016/j.janxdis.2013.01.003 23454838 PMC3628280

[18] Mennin DS, Heimberg RG, Turk CL, Fresco DM. Preliminary evidence for an emotion dysregulation model of generalized anxiety disorder. Behav Res Ther. 2005;43(10):1281–310. 10.1016/j.brat.2004.08.008 16086981

[19] Sighinolfi C, Pala AN, Chiri LR, Marchetti I, Sica C. Difficulties in emotion regulation scale (DERS): The italian translation and adaptation. Psicoter Cogn Comport. 2010;16(2):141–70.

[20] Christophe V, Antoine P, Leroy T, Delelis G. Évaluation de deux stratégies de régulation émotionnelle : la suppression expressive et la réévaluation cognitive. Rev Eur Psychol Appliquée/European Rev Appl Psychol [Internet]. 2009 1 [cited 2017 Oct 4];59(1):59–67. Available from: http://linkinghub.elsevier.com/retrieve/pii/S1162908808000327

[21] Dennis TA. Interactions between emotion regulation strategies and affective style: Implications for trait anxiety versus depressed mood. Motiv Emot [Internet]. 2007 9 24 [cited 2017 Oct 4];31(3):200–7. Available from: http://link.springer.com/10.1007/s11031-007-9069-6

[22] Congard A, Dauvier B, Antoine P, Gilles PY. Integrating personality, daily life events and emotion: Role of anxiety and positive affect in emotion regulation dynamics. J Res Pers [Internet]. 2011;45(4):372–84. Available from: 10.1016/j.jrp.2011.04.004

[23] Enatescu V-R, Enatescu I, Craina M, Gluhovschi A, Papava I, Romosan R, et al State and trait anxiety as a psychopathological phenomenon correlated with postpartum depression in a Romanian sample: a pilot study. J Psychosom Obstet Gynecol [Internet]. 2014 6 14 [cited 2017 Apr 25];35(2):55–61. Available from: http://www.tandfonline.com/doi/full/10.3109/0167482X.2014.914491 10.3109/0167482X.2014.914491 24824599

[24] Kok L, Sep MS, Veldhuijzen DS, Cornelisse S, Nierich AP, van der Maaten J, et al Trait anxiety mediates the effect of stress exposure on post-traumatic stress disorder and depression risk in cardiac surgery patients. J Affect Disord [Internet]. 2016 12 [cited 2017 Sep 29];206:216–23. Available from: http://www.ncbi.nlm.nih.gov/pubmed/27479534 10.1016/j.jad.2016.07.020 27479534

[25] Hovens JE, Op den Velde W, Falger PRJ, De Groen JHM, Van Duijn H. Posttraumatic Stress Disorder in Male and Female Dutch Resistance Veterans of World War II in Relation to Trait Anxiety and Depression. Psychol Rep [Internet]. 1994 2 6 [cited 2017 Sep 29];74(1):275–85. Available from: http://journals.sagepub.com/doi/10.2466/pr0.1994.74.1.275 8153220 10.2466/pr0.1994.74.1.275

[26] Halligan SL, Clark DM, Ehlers A. Cognitive processing, memory, and the development of PTSD symptoms: Two experimental analogue studies. J Behav Ther Exp Psychiatry. 2002;33(2):73–89. 12472172 10.1016/s0005-7916(02)00014-9

[27] Mayou R, Bryant B, Duthie R. Psychiatric consequences of road traffic accidents. Br Med J [Internet]. 1993;307(6905):647–51. Available from: http://www.bmj.com/content/307/6905/647.short 8401049 10.1136/bmj.307.6905.647 PMC1678958

[28] Marzi T, Regina A, Righi S. Emotions shape memory suppression in trait anxiety. Front Psychol [Internet]. 2014 [cited 2017 Sep 29];4:1001 Available from: http://www.ncbi.nlm.nih.gov/pubmed/24427152 10.3389/fpsyg.2013.01001 24427152 PMC3879479

[29] Soeter M, Kindt M. High trait anxiety: a challenge for disrupting fear memory reconsolidation. PLoS One [Internet]. 2013 [cited 2017 Sep 29];8(11):e75239 Available from: http://www.ncbi.nlm.nih.gov/pubmed/24260096 10.1371/journal.pone.0075239 24260096 PMC3832500

[30] Clark IA, Mackay CE, Holmes E a. Low emotional response to traumatic footage is associated with an absence of analogue flashbacks: An individual participant data meta-analysis of 16 trauma film paradigm experiments. Cogn Emot [Internet]. 2014;29(4):702–13. Available from: http://www.ncbi.nlm.nih.gov/pubmed/24920083 10.1080/02699931.2014.926861 24920083 PMC4391283

[31] Wells A, Papageorgiou C. Worry and the incubation of intrusive images following stress. Behav Res Ther. 1995;33(5):579–83. 7598681 10.1016/0005-7967(94)00087-z

[32] Nader K, Schafe GE, Le Doux JE. Fear memories require protein synthesis in the amygdala for reconsolidation after retrieval. Nature [Internet]. 2000 8 17 [cited 2017 Nov 29];406(6797):722–6. Available from: http://www.nature.com/doifinder/10.1038/35021052 10963596 10.1038/35021052

[33] Nader K, Einarsson EÖ. Memory reconsolidation: an update. Ann N Y Acad Sci [Internet]. 2010 Mar 1 [cited 2017 Nov 29];1191(1):27–41. Available from: http://doi.wiley.com/10.1111/j.1749-6632.2010.05443.x 20392274 10.1111/j.1749-6632.2010.05443.x

[34] Ehlers A, Steil R. Maintenance of intrusive memories in posttraumatic stress disorder: A cognitive approach. Behav Cogn Psychother. 1995;23:217–49. 10.1017/S135246580001585X 21241539 PMC2887304

[35] Ehlers A, Clark DM. A cognitive model of posttraumatic stress disorder. Behav Res Ther. 2000;38(4):319–45. 10761279 10.1016/s0005-7967(99)00123-0

[36] Laposa JM, Rector NA. The prediction of intrusions following an analogue traumatic event: Peritraumatic cognitive processes and anxiety-focused rumination versus rumination in response to intrusions. J Behav Ther Exp Psychiatry [Internet]. 2012;43(3):877–83. Available from: http://linkinghub.elsevier.com/retrieve/pii/S0005791611001327 10.1016/j.jbtep.2011.12.007 22296743

[37] Goldsmith RE, Chesney SA, Heath NM, Barlow MR. Emotion regulation difficulties mediate associations between betrayal trauma and symptoms of posttraumatic stress, depression, and anxiety. J Trauma Stress. 2013;26(3):376–84. 10.1002/jts.21819 23737296

[38] James EL, Lau-Zhu A, Clark IA, Visser RM, Hagenaars MA, Holmes EA. The trauma film paradigm as an experimental psychopathology model of psychological trauma: intrusive memories and beyond. Clin Psychol Rev. 2016;47:106–42. 10.1016/j.cpr.2016.04.010 27289421

[39] Olatunji BO, Deacon BJ, Abramowitz JS. The cruelest cure? Ethical issues in the implementation of exposure-based treatments. Cogn Behav Pract [Internet]. 2009;16(2):172–80. Available from: 10.1016/j.cbpra.2008.07.003

[40] Bullinger AH, Rössler A, Müller-Spahn F. From toy to tool: The development of immersive virtual reality environments for psychotherapy of specific phobias In: Riva G, Wiederhold BK, Molinari E, editors. Virtual Environments in Clinical Psychology and Neuroscience. Amsterdam, Netherlands: Ios Press.; 1998 p. 103–11. 10350910

[41] Bordnick PS, Graap KM, Copp HL, Brooks J, Ferrer M. Virtual reality cue reactivity assessment in cigarette smokers. CyberPsychology Behav [Internet]. 2005;8(5):487–92. Available from: http://www.liebertonline.com/doi/abs/10.1089/cpb.2005.8.487 10.1089/cpb.2005.8.487 16232041

[42] Munyan BG, Neer SM, Beidel DC, Jentsch F, Sayer N, Nugent S. Olfactory Stimuli Increase Presence in Virtual Environments. PLoS One [Internet]. 2016 6 16 [cited 2017 Jun 8];11(6):e0157568 Available from: http://dx.plos.org/10.1371/journal.pone.0157568 10.1371/journal.pone.0157568 27310253 PMC4910977

[43] Riva G, Mantovani F, Capideville CS, Preziosa A, Morganti F, Villani D, et al Affective interactions using virtual reality: The link between presence and emotions. Cyberpsychol Behav. 2007;10(1):45–56. 10.1089/cpb.2006.9993 17305448

[44] Kleim B, Westphal M. Mental health in first responders: A review and recommendation for prevention and intervention strategies. Traumatology (Tallahass Fla). 2011;17(4):17–24.

[45] Rovira A, Swapp D, Spanlang B, Slater M. The use of virtual reality in the study of people’s responses to violent incidents. Front Behav Neurosci. 2009;3(December):1–10. 20076762 10.3389/neuro.08.059.2009 PMC2802544

[46] Kinateder M, Ronchi E, Nilsson D, Kobes M, Müller M, Pauli P, et al Virtual reality for fire evacuation research In: Krasuski A, Rein G, editors. Federated Conference on Computer Science and Information Systems. New York: IEEE; 2014 p. 313–21.

[47] Becker-Asano C, Sun D, Kleim B, Scheel CN, Tuschen-Caffier B, Nebel B. Outline of an empirical study on the effects of emotions on strategic behavior in virtual emergencies In: D’Mello S, Graessner A, Schuller B, Martin J-C, editors. Affective Computing and Intelligent Interaction. Berlin Heidelberg: Springer; 2011 p. 508–17.

[48] Spielberger CD, Gorsuch RL, Lushene PR, Vagg PR, Jacobs AG. Manual for the state-trait anxiety inventory (STAI) Palo Alto, CA: Consulting Psychologists Press; 1983.

[49] Sideridis G, Simos P, Papanicolaou A, Fletcher J. Using Structural Equation Modeling to Assess Functional Connectivity in the Brain: Power and Sample Size Considerations. Educ Psychol Meas [Internet]. 2014 10 [cited 2017 Jun 14];74(5):733–58. Available from: http://www.ncbi.nlm.nih.gov/pubmed/25435589 10.1177/0013164414525397 25435589 PMC4245025

[50] Foa EB, Cashman L, Jaycox L, Perry K. The validation of a self-report measure of posttraumatic stress disorder: The Posttraumatic Diagnostic Scale. Psychol Assess [Internet]. 1997 [cited 2017 Oct 10];9(4):445–51. Available from: http://doi.apa.org/getdoi.cfm?doi=10.1037/1040-3590.9.4.445

[51] Leung S-O. A Comparison of Psychometric Properties and Normality in 4-, 5-, 6-, and 11-Point Likert Scales. J Soc Serv Res [Internet]. 2011;37(4):412–21. Available from: http://www.tandfonline.com/doi/abs/10.1080/01488376.2011.580697

[52] Beck AT, Steer RA, Brown GK. Manual for the beck depression inventory-II San Antonio, TX: Psychological Corporation; 1996.

[53] Hautzinger M, Keller F, Kühner C. Das Beck Depressionsinventar II [The Beck Depression Inventory II]. Frankfurt am Main: Harcourt Test Services; 2006.

[54] Gratz KL, Roemer L. Multidimensional assessment of emotion regulation and dysregulation: Development, factor structure, and initial validation of the difficulties in emotion regulation scale. J Psychopathol Behav Assess. 2004;26(1):41–54.

[55] Michael T, Ehlers A. Enhanced perceptual priming for neutral stimuli occurring in a traumatic context: Two experimental investigations. Behav Res Ther. 2007;45(2):341–58. 10.1016/j.brat.2006.03.012 16678789

[56] Boucsein W. Electrodermal Activity 2nd ed New York: Springer; 2012.

[57] Critchley HD. Review: Electrodermal Responses: What Happens in the Brain. Neurosci [Internet]. 2002 4 29 [cited 2017 Jun 8];8(2):132–42. Available from: http://www.ncbi.nlm.nih.gov/pubmed/11954558 10.1177/107385840200800209 11954558

[58] Wilhelm FH, Peyk P. ANSLAB: Autonomic Nervous System Laboratory 4.0 [Computer Software] Basel, Switzerland: Wilhelm & Peyk; 2005.

[59] Hair JF, Black WC, Babin BJ, Anders RE. Multivariate Data Analysis: A Global Perspective 7th ed Upper Saddle River, NJ: Pearson; 2010.

[60] Muthén LK, Muthén BO. Mplus 7.3 [Computer Software] 7th ed Los Angeles, CA: Muthén & Muthén; 2014.

[61] Cohen J. Statistical power analysis for the behavioural sciences 2nd ed Hillsdale, NJ: Lawrence Earlbaum Associates; 1988.

[62] Hu L, Bentler PM. Cutoff criteria for fit indexes in covariance structure analysis: Conventional criteria versus new alternatives. Struct Equ Model a Multidiscip J. 1999;6(1):1–55.

[63] Dretsch MN, Williams K, Emmerich T, Crynen G, Ait-Ghezala G, Chaytow H, et al Brain-derived neurotropic factor polymorphisms, traumatic stress, mild traumatic brain injury, and combat exposure contribute to postdeployment traumatic stress. Brain Behav [Internet]. 2016 1 1 [cited 2017 Sep 29];6(1):e00392 Available from: http://doi.wiley.com/10.1002/brb3.392 27110438 10.1002/brb3.392 PMC4834940

[64] Dretsch M, Thiel K, Athy J, Born S, Prue-Owens K. Posttraumatic Stress Disorder in the U.S. Warfighter: Sensitivity to Punishment and Antidepressant Use Contribute to Decision-Making Performance. Traumatology (Tallahass Fla) [Internet]. 2012 [cited 2017 Sep 29];19(2):118–25. Available from: http://citeseerx.ist.psu.edu/viewdoc/download?doi=10.1.1.864.244&rep=rep1&type=pdf

[65] Dretsch MN, Thiel KJ, Athy JR, Irvin CR, Sirmon-Fjordbak B, Salvatore A. Mood symptoms contribute to working memory decrement in active-duty soldiers being treated for posttraumatic stress disorder. Brain Behav [Internet]. 2012 7 1 [cited 2017 Oct 4];2(4):357–64. Available from: http://doi.wiley.com/10.1002/brb3.53 22950039 10.1002/brb3.53 PMC3432958

[66] Dretsch MN, Wood KH, Daniel TA, Katz JS, Deshpande G, Goodman AM, et al Exploring the Neurocircuitry Underpinning Predictability of Threat in Soldiers with PTSD Compared to Deployment Exposed Controls. Open Neuroimag J [Internet]. 2016 [cited 2017 Sep 29];10:111–24. Available from: http://www.ncbi.nlm.nih.gov/pubmed/27867434 10.2174/1874440001610010111 27867434 PMC5101630

[67] Jezova D, Makatsori A, Duncko R, Moncek F, Jakubek M. High trait anxiety in healthy subjects is associated with low neuroendocrine activity during psychosocial stress. Prog Neuro-Psychopharmacology Biol Psychiatry. 2004;28(8):1331–6. 10.1016/j.pnpbp.2004.08.005 15588760

[68] Wilken JA, Smith BD, Tola K, Mann M. Trait anxiety and prior exposure to non-stressful stimuli: effects on psychophysiological arousal and anxiety. Int J Psychophysiol. 2000;37(3):233–42. 10858569 10.1016/s0167-8760(00)00082-9

[69] Shepherd L, Wild J. Emotion regulation, physiological arousal and PTSD symptoms in trauma-exposed individuals. J Behav Ther Exp Psychiatry [Internet]. 2014;45(3):360–7. Available from: 10.1016/j.jbtep.2014.03.002 24727342 PMC4053589

[70] Cisler JM, Olatunji BO, Feldner MT, Forsyth JP. Emotion regulation and the anxiety disorders: An integrative review. J Psychopathol Behav Assess. 2010;32(1):68–82. 10.1007/s10862-009-9161-1 20622981 PMC2901125

[71] Suveg C, Morelen D, Brewer GA, Thomassin K. The emotion dysregulation model of anxiety: A preliminary path analytic examination. J Anxiety Disord [Internet]. 2010;24(8):924–30. Available from: 10.1016/j.janxdis.2010.06.018 20634040

[72] Eftekhari A, Zoellner LA, Vigil SA. Patterns of emotion regulation and psychopathology. Anxiety Stress Coping. 2009;22(5):571–86. 10.1080/10615800802179860 19381989 PMC3234115

[73] Gross JJ. Emotion regulation: Affective, cognitive, and social consequences. Psychophysiology [Internet]. 2002;39(3):281–91. Available from: http://www.ncbi.nlm.nih.gov/pubmed/12212647 12212647 10.1017/s0048577201393198

[74] Lazarus RS, Folkman S. Stress, Appraisal and Coping New York: Springer Publishing Company; 1984.

[75] Tull MT, Barrett HM, McMillan ES, Roemer L. A preliminary investigation of the relationship between emotion regulation difficulties and posttraumatic stress symptoms. Behav Ther. 2007;38(3):303–13. 10.1016/j.beth.2006.10.001 17697854

[76] Shalev A, Peri TP, Canetti L, Schreiber S. Predictors of PTSD in injured trauma survivors: A prospective study. Am J Psychiatry,. 1996;153(2):219–25. 10.1176/ajp.153.2.219 8561202

[77] Michael T, Ehlers A, Halligan SL, Clark DM. Unwanted memories of assault: What intrusion characteristics are associated with PTSD? Behav Res Ther. 2005;43(5):613–28. 10.1016/j.brat.2004.04.006 15865916

[78] Reynolds M, Brewin CR. Intrusive memories in depression and posttraumatic stress disorder. Behav Res Ther. 1999;37(3):201–15. 10087639 10.1016/s0005-7967(98)00132-6

[79] Bishop SJ. Trait anxiety and impoverished prefrontal control of attention. Nat Neurosci [Internet]. 2009 1 14 [cited 2017 Sep 29];12(1):92–8. Available from: http://www.nature.com/doifinder/10.1038/nn.2242 19079249 10.1038/nn.2242

[80] Myers LB. Repressive coping, trait anxiety and reported avoidance of negative thoughts. Pers Individ Dif [Internet]. 1998 3 [cited 2016 Jul 4];24(3):299–303. Available from: http://linkinghub.elsevier.com/retrieve/pii/S0191886997001803

[81] Clark DA, Hemsley DR. Individual differences in the experience of depressive and anxious, intrusive thoughts. Behav Res Ther [Internet]. 1985 1 [cited 2016 Apr 13];23(6):625–33. Available from: http://www.sciencedirect.com/science/article/pii/0005796785900579 4074275 10.1016/0005-7967(85)90057-9

[82] Ehlers A, Mayou RA, Bryant B. Psychological predictors of chronic posttraumatic stress disorder after motor vehicle accidents. J Abnorm Psychol [Internet]. 1998 [cited 2016 Jun 23];107(3):508–19. Available from: http://doi.apa.org/getdoi.cfm?doi=10.1037/0021-843X.107.3.508 9715585 10.1037//0021-843x.107.3.508

[83] Mennin DS, Holaway RM, Fresco DM, Moore MT, Heimberg RG. Delineating components of emotion and its dysregulation in anxiety and mood psychopathology. Behav Ther. 2007;38(3):284–302. 10.1016/j.beth.2006.09.001 17697853

[84] Stevens NR, Gerhart J, Goldsmith RE, Heath NM, Chesney SA, Hobfoll SE. Emotion Regulation Difficulties, Low Social Support, and Interpersonal Violence Mediate the Link Between Childhood Abuse and Posttraumatic Stress Symptoms. Behav Ther. 2013;44(1):152–61. 10.1016/j.beth.2012.09.003 23312434

[85] Rose Barlow M, Goldsmith Turow RE, Gerhart J. Trauma appraisals, emotion regulation difficulties, and self-compassion predict posttraumatic stress symptoms following childhood abuse. Child Abuse Negl [Internet]. 2017 [cited 2017 Nov 29];65:37–47. Available from: https://ac.els-cdn.com/S0145213417300054/1-s2.0-S0145213417300054-main.pdf?_tid=9694cabe-d4e7-11e7-b08a-00000aab0f02&acdnat=1511947859_d26ed6290c520feaedabb40c67cdf098 10.1016/j.chiabu.2017.01.006 28110110

[86] Greening SG, Mitchell DGV. A network of amygdala connections predict individual differences in trait anxiety. Hum Brain Mapp [Internet]. 2015 12 1 [cited 2017 Nov 29];36(12):4819–30. Available from: http://doi.wiley.com/10.1002/hbm.22952 26769550 10.1002/hbm.22952 PMC6869108

[87] Kim MJ, Whalen PJ. The structural integrity of an amygdala-prefrontal pathway predicts trait anxiety. J Neurosci [Internet]. 2009 9 16 [cited 2017 Nov 29];29(37):11614–8. Available from: http://www.ncbi.nlm.nih.gov/pubmed/19759308 10.1523/JNEUROSCI.2335-09.2009 19759308 PMC2791525

[88] Ochsner KN, Ray RD, Cooper JC, Robertson ER, Chopra S, Gabrieli JDE, et al For better or for worse: neural systems supporting the cognitive down- and up-regulation of negative emotion. Neuroimage [Internet]. 2004;23(2):483–99. Available from: http://linkinghub.elsevier.com/retrieve/pii/S1053811904003404 10.1016/j.neuroimage.2004.06.030 15488398

[89] Schartau PES, Dalgleish T, Dunn BD. Seeing the bigger picture: Training in perspective broadening reduces self-reported affect and psychophysiological response to distressing films and autobiographical memories. J Abnorm Psychol [Internet]. 2009 2 [cited 2017 Nov 29];118(1):15–27. Available from: http://www.ncbi.nlm.nih.gov/pubmed/19222310 10.1037/a0012906 19222310

[90] Elwood LS, Hahn KS, Olatunji BO, Williams NL. Cognitive vulnerabilities to the development of PTSD: A review of four vulnerabilities and the proposal of an integrative vulnerability model. Clin Psychol Rev. 2009;29(1):87–100. 10.1016/j.cpr.2008.10.002 19008028

[91] McKeever VM, Huff ME. A diathesis-stress model of posttraumatic stress disorder: Ecological, biological, and residual stress pathways. Rev Gen Psychol. 2003;7(3):237–50.

[92] Davies MI, Clark DM. Predictors of analogue post-traumatic intrusive cognitions. Behav Cogn Psychother [Internet]. 1998 [cited 2016 Mar 18];26(4):303–14. Available from: http://journals.cambridge.org/download.php?file=%2FBCP%2FBCP26_04%2FS135246589826402Xa.pdf&code=9c979a82cb791fcf6798fcc5c0bfb3b2

[93] Mineka S, Watson D, Clark LA. Comorbidity of anxiety and unipolar mood disorders. Annu Rev Psychol [Internet]. 1998 1 28 [cited 2016 Jun 1];49:377–412. Available from: http://www.annualreviews.org/doi/abs/10.1146/annurev.psych.49.1.377 9496627 10.1146/annurev.psych.49.1.377

[94] Reynolds M, Salkovskis PM. The relationship among guilt, dysphoria, anxiety and obsessions in a normal population—An attempted replication. Behav Res Ther [Internet]. 1991 1 [cited 2016 Jun 1];29(3):259–65. Available from: http://www.sciencedirect.com/science/article/pii/000579679190116K 1883306 10.1016/0005-7967(91)90116-k

[95] Aldao A, Nolen-Hoeksema S, Schweizer S. Emotion-regulation strategies across psychopathology: A meta-analytic review. Clin Psychol Rev. 2010;30(2):217–37. 10.1016/j.cpr.2009.11.004 20015584

[96] García-Campayo J, del Hoyo YL, Valero MS, Yus MCP, Esteban EA, Guedea MP, et al Primary prevention of anxiety disorders in primary care: A systematic review. Prev Med (Baltim). 2015;76:S12–5. 10.1016/j.ypmed.2014.10.015 25456801

[97] Bonanno GA, Pat-Horenczyk R, Noll J. Coping flexibility and trauma: The Perceived Ability to Cope With Trauma (PACT) scale. Psychol Trauma Theory, Res Pract Policy [Internet]. 2011;3(2):117–29. Available from: http://doi.apa.org/getdoi.cfm?doi=10.1037/a0020921

[98] Rodin R, Bonanno GA, Knuckey S, Satterthwaite ML, Hart R, Joscelyne A, et al Coping flexibility predicts post-traumatic stress disorder and depression in human rights advocates. Int J Ment Health [Internet]. 2017 7 18 [cited 2017 Oct 12];1–12. Available from: https://www.tandfonline.com/doi/full/10.1080/00207411.2017.1345047

[99] Cheng C, Kogan A, Chio JH. The effectiveness of a new, coping flexibility intervention as compared with a cognitive-behavioural intervention in managing work stress. Work Stress. 2012;26(3):272–88.

[100] Shipherd JC, Salters-Pedneault K, Fordiani J, Fordiani J. Evaluating postdeployment training for coping with intrusive cognition: A comparison of training approaches. J Consult Clin Psychol. 2016;84(11):960–71. 10.1037/ccp0000136 27599228

