# Supplementary figures and images for: The impact of pre-existing anxiety on affective and cognitive processing of a Virtual Reality analogue trauma
Source: PLoS One. 2017 Dec 29;12(12):e0190360. doi: 10.1371/journal.pone.0190360 (PMC5747458; doi:10.1371/journal.pone.0190360)

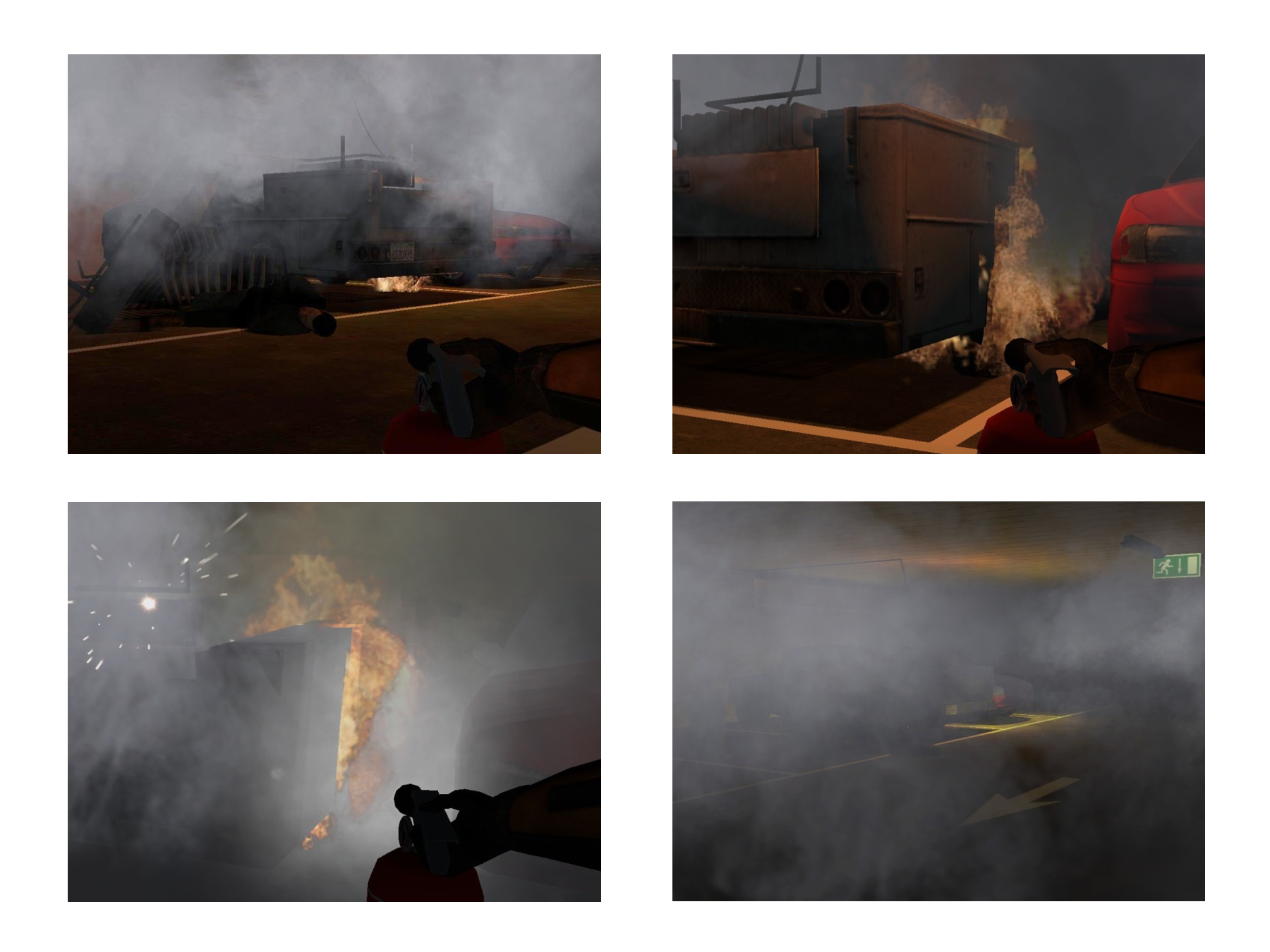

Supplement: S1 Fig — (TIF) [file pone.0190360.s001.tif]
