# Supplementary material for: The impact of pre-existing anxiety on affective and cognitive processing of a Virtual Reality analogue trauma
Source: PLoS One. 2017 Dec 29;12(12):e0190360. doi: 10.1371/journal.pone.0190360 (PMC5747458; doi:10.1371/journal.pone.0190360)
Supplement: S1 Table — Note. t1 = 5 min after the VR analogue trauma; t2 = 9 pm the next day. Combined items of the modified version of the intrusive memory questionnaire (IMQ): (i) frequency of pictures and sounds, (ii) temporal occurrence of pictures and sounds to group sensory vs. cognitive modalities; (iii) worry about intrusive pictures, thoughts and sounds as subsequent appraisal. (PDF) [file pone.0190360.s002.pdf]

	Trait anxiety	
	low	high
	M (SD)	*M (SD)*
Subjective stress response		
Anxiety Baseline	1.47 (2.19)	1.76 (2.34)
Anxiety Analogue trauma	6.21 (2.41)	7.44 (2.16)
Anxiety Recovery	1.72 (2.13)	2.96 (2.35)
Physiological stress response		
Skin conductance level Baseline	1.418 (0.654)	1.627 (0.916)
Skin conductance level Analogue trauma	3.461 (1.834)	4.076 (2.033)
Skin conductance level Recovery	3.247 (1.931)	3.710 (2.197)
Emotional dysregulation		
Non-acceptance	10.53 (3.21)	14.55 (4.91)
Goals	10.55 (3.34)	14.68 (4.85)
Impulse	8.25 (2.36)	11.15 (4.96)
Awareness	12.80 (3.58)	14.65 (4.87)
Strategies	11.58 (3.62)	21.03 (7.26)
Clarity	8.00 (2.14)	11.90 (4.46)
Intrusive memories		
Frequency of pictures/sounds t1	3.18 (2.44)	4.56 (3.52)
Frequency of pictures/sounds t2	1.08 (1.16)	1.67 (2.03)
Frequency of thoughts t1	3.00 (2.26)	4.54 (3.33)
Frequency of thoughts t2	1.59 (2.37)	2.28 (2.99)
Temporal occurrence of pictures/sounds t1	4.26 (2.74)	5.67 (2.46)
Temporal occurrence of pictures/sounds t2	1.74 (1.48)	2.05 (1.23)
Temporal occurrence of thoughts t1	4.10 (2.59)	5.64 (2.75)
Temporal occurrence of thoughts t2	1.62 (0.82)	2.10 (1.31)
General mental occupation t1	5.62 (2.92)	6.97 (2.99)
General mental occupation t2	1.92 (1.16)	2.49 (1.60)
Perceived worry t1	4.54 (2.59)	6.08 (2.75)
Perceived worry t2	1.62 (1.25)	3.13 (2.41)
